# High Efficiency Lipid-Based siRNA Transfection of Adipocytes in Suspension

**DOI:** 10.1371/journal.pone.0006940

**Published:** 2009-09-11

**Authors:** Gail Kilroy, David H. Burk, Z. Elizabeth Floyd

**Affiliations:** 1 Ubiquitin Biology Laboratory, Pennington Biomedical Research Center, Baton Rouge, Louisiana, United States of America; 2 Cell Biology and Bioimaging Core, Pennington Biomedical Research Center, Baton Rouge, Louisiana, United States of America; University of Hong Kong, China

## Abstract

**Background:**

Fully differentiated adipocytes are considered to be refractory to introduction of siRNA via lipid-based transfection. However, large scale siRNA-based loss-of-function screening of adipocytes using either electroporation or virally-mediated transfection approaches can be prohibitively complex and expensive.

**Methodology/Principal Findings:**

We present a method for introducing small interfering RNA (siRNA) into differentiated 3T3-L1 adipocytes and primary human adipocytes using an approach based on forming the siRNA/cell complex with the adipocytes in suspension rather than as an adherent monolayer, a variation of “reverse transfection”.

**Conclusions/Significance:**

Transfection of adipocytes with siRNA by this method is economical, highly efficient, has a simple workflow, and allows standardization of the ratio of siRNA/cell number, making this approach well-suited for high-throughput screening of fully differentiated adipocytes.

## Introduction

Murine 3T3-L1 adipocytes are a well-characterized cell culture model that is widely used to study the role of adipocyte biology in obesity and type 2 diabetes. These properties make 3T3-L1 adipocytes an attractive model for carrying out loss-of-function assays using siRNA technology. However, fully differentiated 3T3-L1 adipocytes are among the most difficult cell types to transfect efficiently with siRNA using standard lipid-based techniques. Typically, siRNA is introduced into 3T3-L1 adipocytes using either electroporation or virally-mediated approaches [1], [2], [3], [4]. Both of these approaches have limitations in systematic siRNA-mediated screening experiments, including the potential cell damage and equipment and reagent costs associated with electroporation in a high-throughput format or the complexity and safety issues associated with virally-mediated transfection. Alternatives include peptide-based transfection reagents that are highly efficient, but require sonication of the peptide prior to transfection and have not been demonstrated in fully differentiated adipocytes [5]. “Reverse transfection”, also known as solid phase optimized transfection RNAi (SPOT-RNAi) [6], is an alternative that uses glass plates or cell culture plates preloaded with siRNA and to which the cells of interest are then added.

With improved transfection efficiency, lipid-based siRNA transfection using a version of “reverse transfection” in which the siRNA and cells are mixed in suspension would offer the simplest and least expensive approach to systematic screening using siRNA in adipocytes. The adipocytes would then be allowed to reattach to an adherent plate surface while in the presence of the siRNA complex. This approach has been reported in the human melanoma cell line LOX, another cell line that is considered difficult to transfect using lipid-based reagents [7].

Herein, we present a method for lipid-mediated siRNA transfection of fully differentiated 3T3-L1 adipocytes and primary human adipocytes that is based on incubating the siRNA/lipid complex with the detached adipocytes in suspension. This results in highly efficient siRNA transfection and is simple, cost-effective, and nontoxic, making this approach well suited to systematic high throughput siRNA screening of fully differentiated adipocytes.

## Materials and Methods

### Materials

Dulbecco's Modified Eagle's Media (DMEM) was purchased from MediaTech. Bovine and fetal bovine (FBS) serums were obtained from Hyclone. Insulin, IBMX, dexamethasone , DAPI, and collagen (#C-7116) were purchased from Sigma-Aldrich. OptiMEM, Calcein-AM, and propidium iodide were from Invitrogen. The mouse monoclonal PPARγ (sc-7273), goat polyclonal lamin A/C (sc-6215), and mouse monoclonal β-actin (sc-47778) antibodies were purchased from Santa Cruz Biotechnology. The rabbit polyclonal E6-AP antibody (A300–352A) was from Bethyl Laboratories. Horseradish peroxidase conjugated secondary antibodies were obtained from Jackson Immunoresearch Laboratories. DharmaFECT transfection reagents, siGLO RISC-free labeled with DY-547 (Rhodamine filter) siRNA, siGenome non-targeting siRNA Pool #2, lamin A/C siRNA, TBL-1 and TBLR-1 siRNA were purchased from Thermo Fisher Scientific-Dharmacon. E6-AP and PPARγ siRNAs were purchased from Santa Cruz Biotechnology. Polyfect was purchased from Qiagen and Xfect was purchased from Clontech. All TaqMan primer/probes pairs were obtained from Applied Biosystems. Differentiated human primary adipocytes (lot # L041806) were purchased from Zen-Bio, Inc.

### Cell Culture

Murine 3T3-L1 preadipocytes were cultured in Dulbecco's modified Eagle's medium (DMEM) high glucose containing 10% calf serum and antibiotics (100 units/ml penicillin G and 100 µg/ml streptomycin). To obtain fully differentiated adipocytes, the 3T3-L1 preadipocytes were plated and grown on 10 cm plates to 2 days post confluence and induced to differentiate using a standard induction cocktail of 3-isobutyl-1-methylxanthine, dexamethasone, and insulin (MDI) as previously described [8]. After 48 hours this medium was replaced with DMEM high glucose supplemented with 10% fetal bovine serum (FBS) and the cells were maintained in this medium. The 3T3-L1 adipocytes were used for transfection at day 4-6 post-induction when lipid droplets were readily apparent.

The differentiated adipocytes were rinsed in phosphate-buffered saline (PBS, 5 ml) prewarmed to 37°C and detached from the plate via trypsin treatment (1 ml of 0.25% trypsin/10 cm plate) at 37°C. The adipocytes were in contact with the trypsin only until the cells began to detach, approximately 2–5 minutes. The detached adipocytes were gently resuspended in DMEM, high glucose with 10% FBS and antibiotics (5 ml) and collected by centrifugation at 514×*g* (1000 rpm, Beckman Coulter GH 3.8 rotor) for five minutes at room temperature. The pelleted adipocytes were gently resuspended in DMEM, high glucose with 10% FBS and antibiotics (5 ml) and an aliquot was counted using a hemocytometer after the addition of trypan blue. A typical yield was 1.0−1.2×10^7^ cells/plate.

### Optimization of siRNA Transfection of 3T3-L1 Adipocytes

To determine optimal conditions for lipid-based siRNA transfection of the adipocytes, we established a grid in the 48 well format to test four variables concurrently: cell density, transfection reagent, siRNA concentration, and transfection reagent volume. Each grid allows optimization using two cell densities, two concentrations of siRNA, and four different transfection reagents at three volumes each. The plates were collagen-coated throughout these experiments. The adipocytes were replated at 5.4×10^4^ cells/cm^2^ or 1.16×10^5^ cells/cm^2^ in the 48 well format at 1 cm^2^/well. We initially used several lipid-based transfection reagents including DharmaFECT 4, DharmaFECT Duo, Polyfect, and Xfect. Each transfection reagent was tested at 0.7 µl/cm^2^, 1.4 µl/cm^2^, or 3.7 µl/cm^2^. At 3.7 µl/cm^2^ of the transfection reagents, the cells detached from the plate within 24 hours. Therefore subsequent experiments were carried out with DharmaFECT D4, DharmaFECT Duo, Polyfect, or Xfect at 0.7 µl/cm^2^ or 1.4 µl/cm^2^ with optimal results obtained using DharmaFECT Duo at 1.4 µl/cm^2^. During optimization, fluorescently labeled siRNA that does not induce the RNA silencing complex (RISC-free) was used at 25 nM or 100 nM for the siRNA complex. The siRNA complex was formed in the 48 well plate by adding equal volumes of the stock concentration of siRNA and OptiMEM and incubating at room temperature in a laminar flow hood. At the end of 5 minutes, the transfection reagent was added along with additional OptiMEM to yield a final total volume of 40 µl/well and incubated at room temperature. At the end of 20 minutes, the resuspended adipocytes at the desired concentration in a total volume of 200 µl were added and the siRNA complex: adipocytes mixture was plated and incubated at 37°C, 5% CO_2_ for 24 hours before the media was exchanged for DMEM, high glucose with 10% FBS and antibiotics.

### Detection of Fluorescent Labeled siRNA and DAPI Stained Nuclei

At 24 and 48 hours post-transfection, transfection efficiency was assayed via fluorescent detection of the labeled siRNA using a Nikon Eclipse TS100 inverted microscope equipped with rhodamine and DAPI filters and Metamorph version 6.1 software. DAPI staining was used to determine the cellular location of the transfected siRNA. Adipocytes transfected with fluorescent labeled [DY-547 (Rhodamine filter)] RISC-free siRNA were incubated with DAPI (0.14 mM) in media for 2.5 hours at 37°C, 5% CO_2_ without fixation of the cells. Thereafter, the adipocytes were rinsed twice with media and the DAPI signal was visualized. Merged images were generated using ImageJ software.

### Classical Transfection of 3T3-L1 adipocytes with siRNA

3T3-L1 preadipocytes were plated at 1.165×10^5^ cells/cm^2^ in a 48 well plate format and induced to differentiate 24 hours later using the standard induction cocktail as described under Cell Culture. The adipocytes were transfected with 25 nM or 100 nM siRNA and 1.4 µl/cm^2^ DharmaFECT Duo for each well at days 4 or 5 post-induction. Transfection efficiency was assessed 48 hours post-transfection by detection of fluorescent labeled RISC-free siRNA and western blot analysis of siRNA targeted proteins.

### Transfection of Human Primary Adipocytes with siRNA in Suspension

Primary human subcutaneous adipocytes were obtained at day 7 post induction and were maintained in DMEM/F-12 with 3% FBS, 33 µM biotin, 17 µM pantothenate, 1 µM bovine insulin, 1 µM dexamethasone, and 100 U penicillin/100 µg streptomycin/0.25 µg Fungizone. At day 9 post-induction, the adipocytes were transfected in suspension as described for the 3T3-L1 adipocytes using 1.165×10^5^ cells/25 nM siRNA or 1.165×10^5^ cells/100 nM siRNA and plated on collagen-coated 48 well plates at 4.1×10^4^ cells/cm^2^. Transfection efficiency was assessed 48 hours post-transfection by detection of fluorescent labeled RISC-free siRNA and western blot analysis of siRNA targeted proteins.

### Cell Viability Assay

Cell viability was determined using calcein-acetoxymethyl (calcein-AM) to stain viable cells and DNA intercalation of propidium iodide (PI) to stain dead cells. The 3T3-L1 adipocytes at 5.4×10^4^ or 1.16×10^5^ cells/cm^2^ were transfected with non-targeting siRNA (25 nM and 100 nM of siGenome non-targeting siRNA, pool #2, which includes luciferase siRNA) and incubated for 48 hours with a single media change at 24 hours after transfection. The cells were then rinsed twice with PBS and incubated with calcein-AM (10 µM) and PI (10 µM) in PBS at 37°C for 15 minutes. The fluorescent signals were detected and quantitated using a Flexstation 2 fluorometer (Molecular Devices) and Softmax Pro 4.8 software at an excitation of 490 nm and emission of 515 nm for detection of fluorescence generated from the calcein-AM and an excitation of 535 nm and emission of 617 nm for detection of PI bound to DNA.

### Real-time RT-PCR

**Figure 1:**
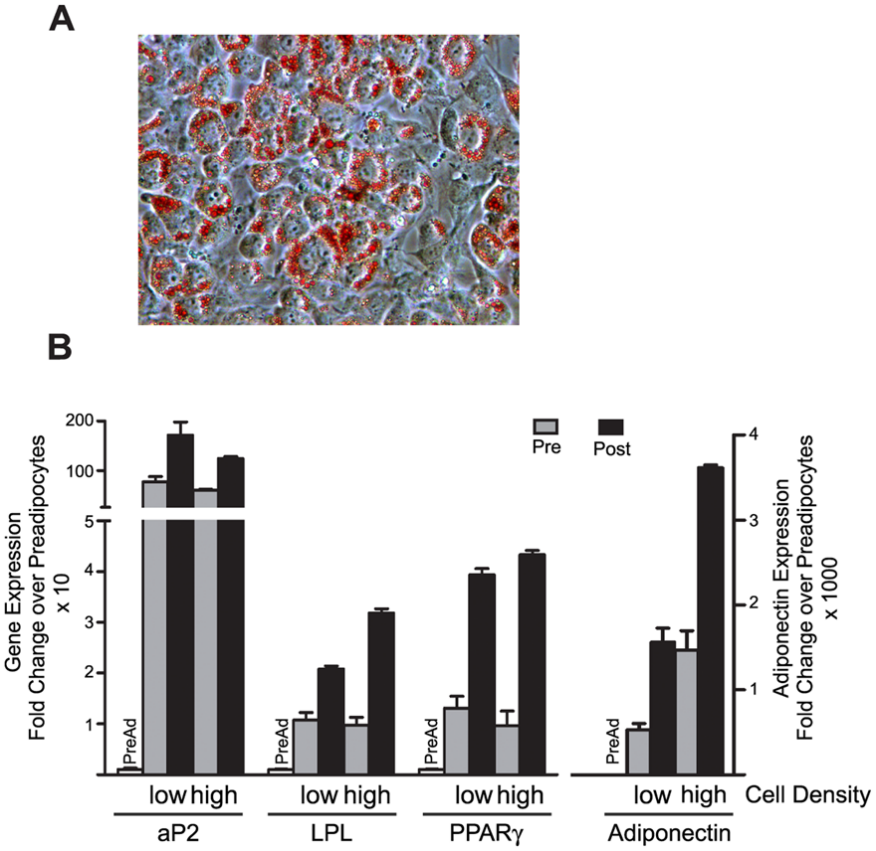
Adipocytes continue to differentiate after harvesting and replating post-induction. The 3T3-L1 preadipocytes were induced to undergo adipogenesis and harvested at day 3–4 post induction when lipid droplets were clearly visible. (A) Oil Red O staining of neutral lipids forty-eight hours after harvesting and replating. (B) The gene expression of adipocyte marker genes aP2, LPL, PPARγ, and adiponectin was measured via real-time PCR upon harvesting (Pre) and forty eight hours after (Post) the adipocytes were replated at 5.4×10^4^ cells/cm^2^ (low) or 1.16×10^5^ cells/cm^2^ (high) and compared to the expression of each gene in preadipocytes prior to induction (preAd). Expression of each gene was assayed in triplicate, normalized to cyclophilin B gene expression, and reported as the mean and standard deviation. The results are representative of experiments carried out twice independently.

Total RNA was purified from cultured cells using TriReagent (Molecular Research Center) according to the manufacturer's instructions. When comparing adipocyte marker genes pre and post-replating (Figure 1B) with expression of the marker genes in the preadipocytes, total RNA was purified from equivalent cell numbers harvested prior to replating (“Pre”) and 48 hours after replating (“Post”). RNA (200 ng) was reverse transcribed using Multiscribe Reverse Transcriptase (Applied Biosystems) with random primers at 37°C for 2 hour. Real-time PCR was performed with TaqMan chemistry (Applied Biosystems) using the 7900 Real-Time PCR system (Applied Biosystems) and universal cycling conditions (50°C for 2 minutes; 95°C for 10 minutes; 40 cycles of 95°C for 15 seconds and 60°C for 1 minute; followed by 95°C for 15 seconds, 60°C for 15 seconds and 95°C for 15 seconds). All results were normalized to a *Cyclophilin B* expression control.

### Oil Red O staining

Oil Red O staining was performed as described by Green and Kehinde [9].

### Preparation of Whole Cell Extracts

Cell monolayers were rinsed with phosphate-buffered saline (PBS) and harvested in a denaturing buffer containing 50 mM Tris-Cl, pH 7.4 with 150 mM NaCl, 1 mM EDTA, 1% Igepal, 0.5% Na-deoxycholate, 0.1% SDS, protease inhibitors (1 µM PMSF, 1 µM pepstatin, 50 trypsin inhibitory milliunits of aprotinin, 10 µM leupeptin). Protein concentrations of whole cell extracts were determined using a BCA assay (Thermo Fisher Scientific, Rockford, IL) according to the manufacturer's instructions.

### Gel Electrophoresis and Immunoblotting

Proteins were separated in polyacrylamide (National Diagnostics) gels containing sodium dodecyl sulfate (SDS) according to Laemmli [10] and transferred to nitrocellulose (BioRad) in 25 mM Tris, 192 mM glycine, and 20% methanol. Following transfer, the membrane was blocked in 4% milk for 1 hour at room temperature. The membranes were incubated with mouse monoclonal anti-PPARγ (1∶125 dilution), rabbit polyclonal anti-lamin A/C (1∶200 dilution), or rabbit polyclonal anti-E6-AP (1∶2000 dilution) as indicated for 1–2 hours at room temperature. Following extensive washes, the results were visualized with horseradish peroxidase (HRP)-conjugated secondary antibodies and enhanced chemiluminescence (Pierce).

## Results and Discussion

We first confirmed that the fully differentiated adipocytes could be replated after trypsinization in a 48 well format and continue to express adipocyte markers. As shown in Figure 1A, when harvested and replated on day 4 post-induction, the adipocytes continue to accumulate neutral lipids when assayed using Oil Red O staining (**Figure 1A**) 48 hours after replating on the collagen-coated plates. When replated at 5.8×10^4^ cells/cm^2^ (“low” cell density, **Figure 1B**) or 1.16×10^5^ cells/cm^2^ (“High” cell density, **Figure 1B**), there is increased gene expression of lipoprotein lipase (LPL), fatty acid binding protein (aP2), peroxisome proliferator activated receptor gamma (PPARγ), and adiponectin (**Figure 1B**) when compared to the levels of each gene present in the preadipocytes, indicating the adipocytes continue to undergo differentiation after harvesting and re-adherence to the collagen-coated plates. LPL and adiponectin were significantly increased at a cell density of 1.16×10^5^ cells/cm^2^ (“High”) compared to a cell density of 5.8×10^4^ cells/cm^2^ (“low”).

**Figure 2:**
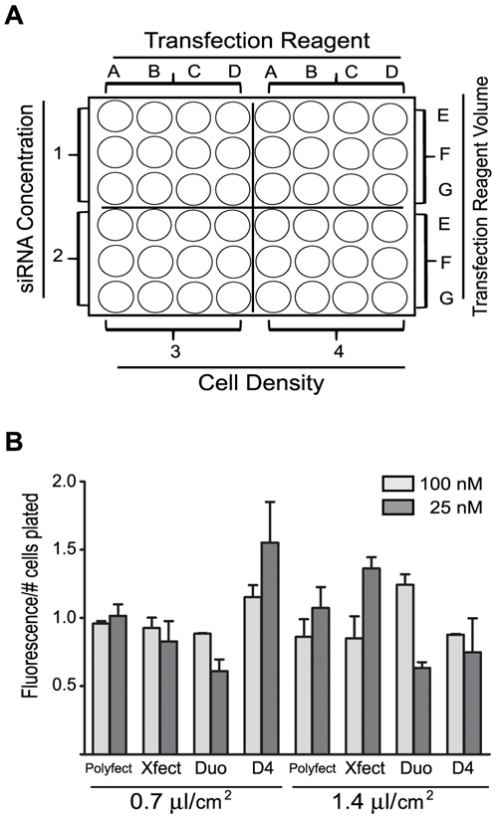
Optimization of siRNA transfection of adipocytes in suspension. (A) Grid layout for testing transfection variables based on a 48 well plate format. This grid accommodates testing two concentrations of siRNA (1,2) when transfecting cells at two densities/cm^2^ (3,4) with four transfection reagents (A,B,C,D) at three concentrations each (E, F, G). (B) Maximal fluorescent signal/cell number plated was obtained with three transfection reagents (D4, Duo, Xfect) at either 25 nM siRNA (D4 or Xfect) or 100 nM siRNA (Duo). The siRNA is siGLO RISC-free labeled with DY-547 (rhodamine filter). The cells were plated at 1.16×10^5^ cells/cm^2^. The fluorescent signals were detected and quantitated using a Flexstation 2 fluorometer (Molecular Devices) and Softmax Pro 4.8 software. The fluorescent signal was assayed in triplicate, normalized to the number of cells plated/well, and reported as the mean and standard deviation from experiments carried out twice independently.

**Figure 3:**
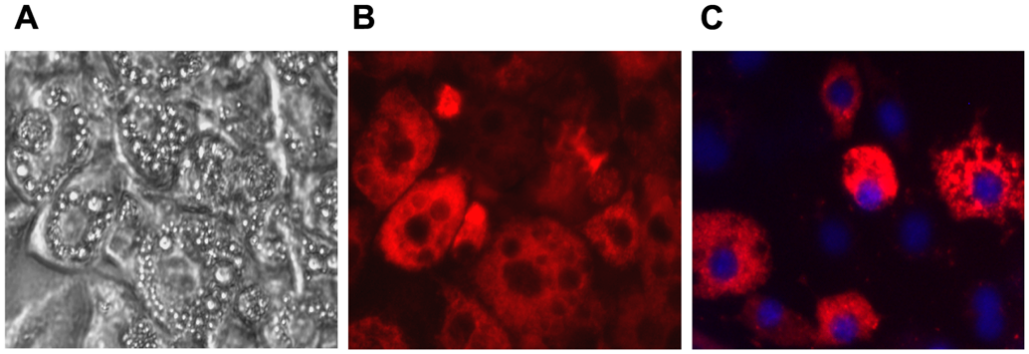
Adipocytes in suspension are efficiently transfected with siRNA. Uptake of the fluorescent-labeled siRNA was assayed at 48 hours post transfection of adipocytes plated at 1.16×10^5^ cells/cm^2^. (A) Brightfield and (B) fluorescent (rhodamine filter) image of the transfected cells taken with a 40X objective. (C) Co-staining with DAPI and the fluorescent-labeled siRNA indicates the siRNA is located in the cytoplasm. The image in (C) is from a different experiment than the paired images in (A) and (B). This experiment was carried out independently greater than four times.

We then used a 48 well grid (**Figure 2A**) to test a combination of factors, including cell density, transfection reagent, transfection reagent volume, siRNA concentration, and incubation time, to determine if the adipocytes could be efficiently transfected in suspension using lipid-based reagents. We initially assessed the fluorescence signal obtained with each transfection reagent (Figure 2B). Maximal fluorescent signal/cell number plated was obtained with three transfection reagents (D4, Duo, Xfect) at either 25 nM siRNA (D4 or Xfect) or 100 nM siRNA (Duo). The optimal combination of transfection efficiency and cell viability in the fully differentiated adipocytes was obtained using 1.16×10^5^ cells/cm^2^, 100 nM siRNA, and 1.4 µl/cm^2^ DharmaFECT Duo. As shown in Figure 3, the fluorescent-tagged siRNA is localized in the adipocytes and appears to be excluded from the lipid droplets. Co-staining with DAPI indicates that the siRNA is localized to the cytoplasm (**Figure 3C**).

As an example of our approach, to test the efficiency of transfection using 1.16×10^5^ cells/cm^2^, 1.4 µl of DharmaFECT Duo/cm^2^, and 100 nM siRNA, for each well of the 48 well plate, the siRNA complex was formed by combining 10 µl of OptiMEM and 10 µl of 2 µM siRNA (in H_2_O) at room temperature. After 5 minutes, 18.6 µl OptiMEM was added followed by 1.4 µl DharmaFECT Duo and incubated at room temperature. At the end of 20 minutes, 200 µl of adipocytes resuspended in media at 5.8×10^5^ cells/ml was added to obtain 1.16×10^5^ cells/cm^2^ (for 1 cm^2^ well).

**Figure 4:**
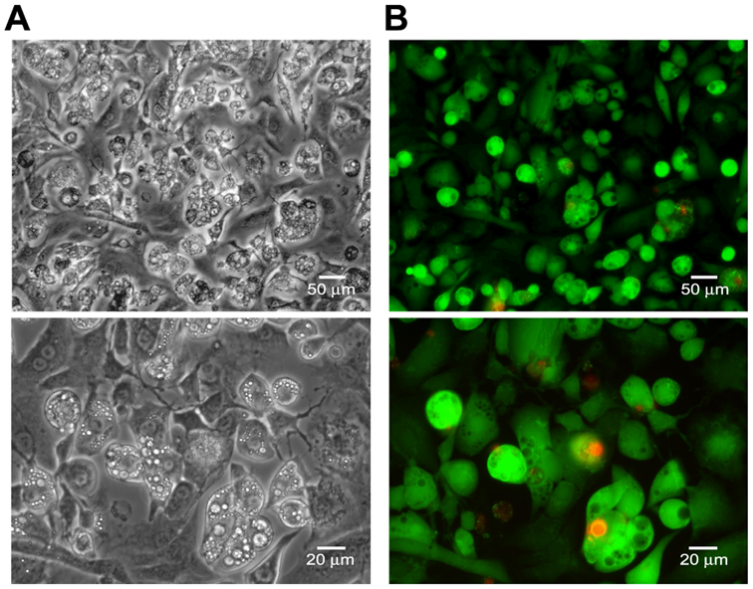
3T3-L1 adipocytes maintain viability with lipid-based siRNA transfection. Cell viability was determined using Calcein-AM (green, viable cells) and propidium iodide (red, non-viable cells) staining in a “live-dead” assay. The assay was carried out at 48 hours post-transfection using non-targeting siRNA (Dharmacon siRNA pool #2 containing luciferase siRNA). (A) Brightfield images taken with a 20X (upper panel) and 40X objective (lower panel). (B) Fluorescent images taken with a 20X (upper panel) and 40X objective (lower panel) showing minimal PI staining. The experiment was carried out twice independently.

When cell viability under the optimized conditions was assayed by the production of calcein from calcein-AM (live cells) or PI binding to DNA (dead cells), the overwhelming majority of adipocytes remain viable compared to the dead cells as assayed using fluorescence detection of calcein and DNA bound propidium iodide (**Figure 4**). This indicates lipid-based siRNA transfection of the adipocytes while in suspension does not adversely affect the viability of fully differentiated adipocytes.

**Figure 5:**
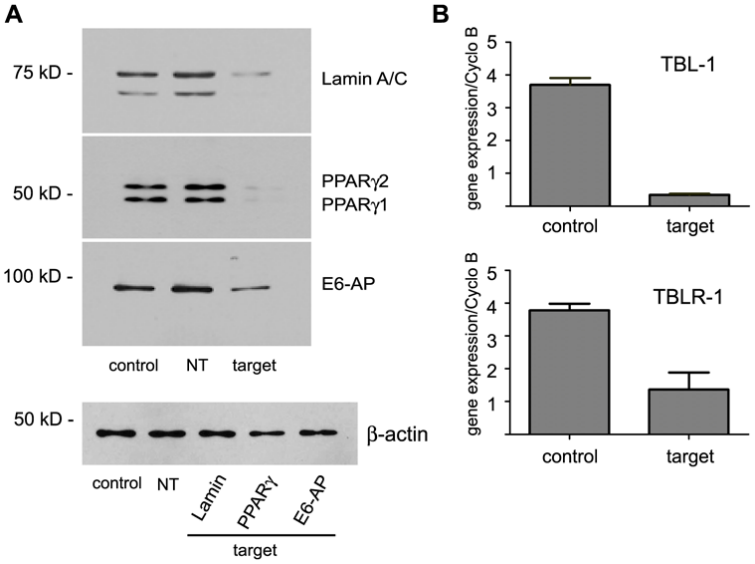
Specific genes are efficiently targeted with lipid-based siRNA transfection of adipocytes. Knockdown of specific genes was assayed at 48 hours post-transfection using either the RISC-free control siRNA (control), nontargeting siRNA (NT), or siRNA targeted to the indicated gene (target). (A) Thirty-five µg of protein was loaded in each lane and separated by SDS-PAGE. Knockdown of lamin A/C, PPARγ1 and PPARγ2, and E6-AP was assayed via western blot analysis. Equal loading of each lane was determined using β-actin expression. (B) knockdown of TBL-1 and TBLR-1 was assayed via real-time PCR in triplicate and reported as the mean and standard deviation. These experiments were carried out independently greater than four times.

The effectiveness of RNAi experiments is determined by the efficiency of siRNA transfection and the ability of the siRNA sequence to silence a specific target mRNA. To test the efficiency of gene knockdown, we assayed the siRNA-mediated decrease in expression of the peroxisome proliferator activated receptor gamma (PPARγ), a nuclear receptor that is required for the formation and maintenance of adipocytes [11]. Along with PPARγ as our gene of interest, we also transfected the fully differentiated adipocytes with siRNA directed against lamin A/C as a control target and the luciferase siRNA (siRNA Pool #2) as a non-targeting control. In addition, we are interested in the role of the ubiquitin proteasome system in regulating PPARγ transcriptional activity in adipocytes. Therefore, we also tested the efficiency of knockdown of three ubiquitin ligases that regulate nuclear hormone receptor activity. E6-AP regulates nuclear receptor activity and targets the estrogen receptor alpha to the proteasome for degradation [12]. The TBL-1 and TBLR-1 genes are ubiquitin ligases that target the nuclear receptor corepressor NCoR to the proteasome for degradation [13], [14]. As shown in **Figure 5**, we obtain efficient knockdown of lamin A/C, PPARγ, and E6-AP proteins when assayed via western blot analysis (**Figure 5A**) and TBL-1 and TBLR-1 when the mRNA levels are assayed via real-time qRT-PCR (**Figure 5B**).

**Figure 6:**
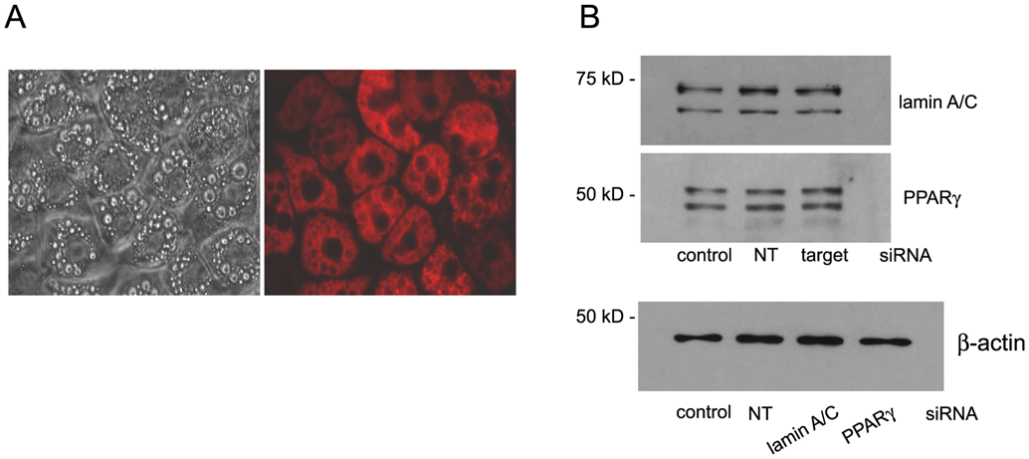
Transfection of adherent 3T3-L1 adipocytes with siRNA does not decrease expression of targeted proteins. Forty-eight hours post-transfection, transfection efficiency was assayed as uptake of fluorescent-labeled siRNA and the siRNA-mediated effect on targeted protein expression. (A) Brightfield and fluorescent (rhodamine filter) images of the transfected cells were taken with a 40X objective. (B) Knockdown of lamin A/C and PPARγ was assayed via western blot analysis post-transfection with either the RISC-free control siRNA (control), nontargeting siRNA (NT), or siRNA targeted to the indicated gene (target). Thirty-five µg of protein was loaded in each lane and separated by SDS-PAGE. Equal loading of each lane was determined using β-actin expression. The experiment was carried out twice independently.

To determine if the siRNA transfection conditions established for adipocytes in suspension would be applicable to adherent adipocytes, we compared siRNA transfection of the 3T3-L1 adipocytes in suspension with the classical approach of introducing siRNA into cells that remain attached (Figure 6). While the fluorescent-tagged RISC-free siRNA is clearly present in the adipocytes (Figure 6A), there was no decrease in the level of the targeted proteins, including PPARγ as an adipocyte specific target (Figure 6B). The lack of change in target protein expression is surprising, given the efficiency of introducing the fluorescent tagged RISC-free siRNA into the adherent adipocytes. However, this result suggests that incubation of the siRNA complex with the adipocytes in suspension or the process of adipocyte readherence in the presence of a targeting siRNA complex may enhance induction of the RNAi silencing complex (RISC) response or delivery of the siRNA to RISC in the adipocytes.

**Figure 7:**
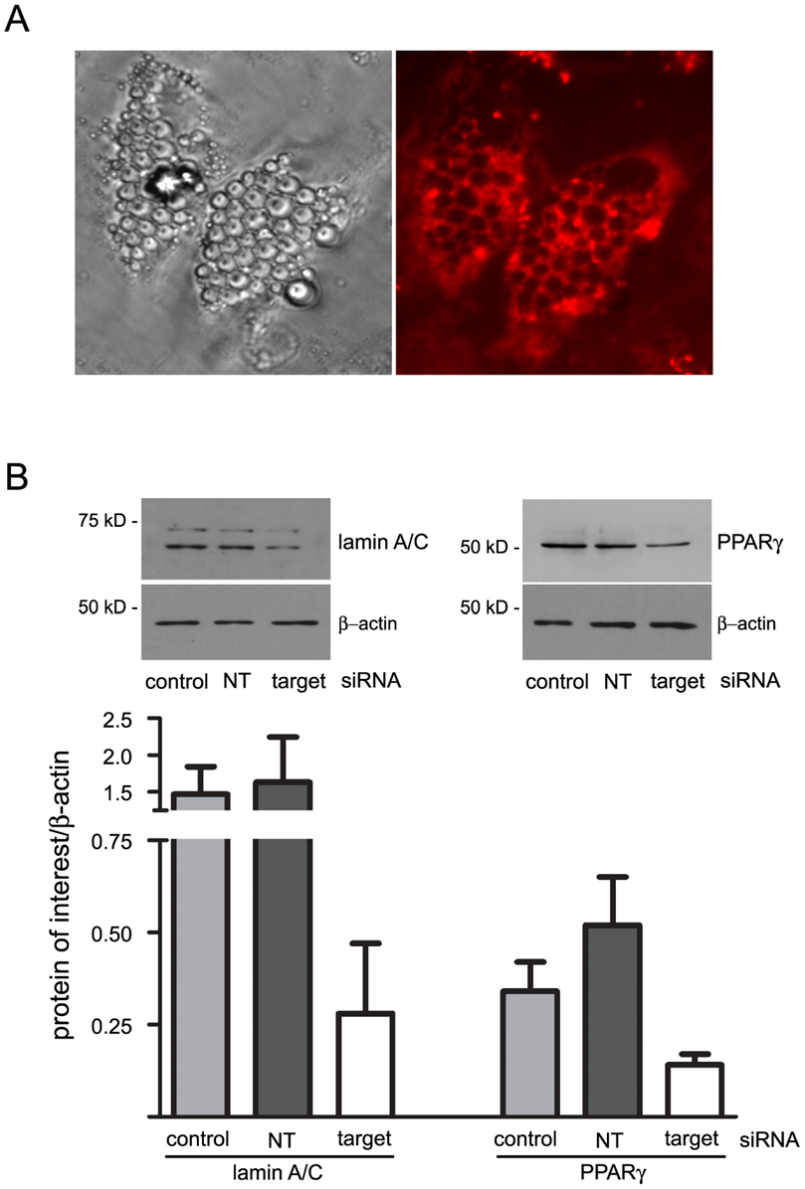
Transfection of human primary adipocytes in suspension with siRNA is associated with decreased expression of targeted proteins. The human adipocytes were transfected on day 9 post-induction (1.165×10^5^ cells/100 nM siRNA ) and plated at 4.1×10^4^ cells/cm^2^. Forty-eight hours post-transfection, transfection efficiency was assayed as uptake of fluorescent-labeled siRNA and the siRNA-mediated effect on targeted protein expression. (A) Brightfield and fluorescent (rhodamine filter) images of the transfected cells were taken with a 40X objective. (B) Knockdown of lamin A/C and PPARγ was assayed via western blot analysis post-transfection with either the RISC-free control siRNA (control), nontargeting siRNA (NT), or siRNA targeted to the indicated gene (target). Thirty-five µg of protein was loaded in each lane and separated by SDS-PAGE. Equal loading of each lane was determined using β-actin expression. The mean and standard deviation of the ratio lamin A/C and PPARγ compared to β-actin was determined after the expression levels of each protein were quantified using Un-Scan-It software (version 6.1) from samples run in triplicate. The experiment was carried out twice independently.

To extend our method to a model of human adipocytes, we applied the optimized (1.165×10^5^ cells/100 nM siRNA) conditions for the 3T3-L1 adipocytes to fully differentiated primary human adipocytes cultured from subcutaneous adipose tissue (Figure 7). The fluorescent-tagged siRNA is apparent in the human adipocytes and is excluded from the lipid droplets (Figure 7A) as occurs with the 3T3-L1 adipocytes (Figure 3A,B). Western blot analysis shows that siRNA targeting lamin A/C and PPARγ reduces the expression of both proteins (Figure 7B). While further optimization is expected to improve the efficiency of gene knockdown, PPARγ protein expression is reduced to approximately 40% of the control levels and lamin A/C protein expression is reduced to approximately 20% of the control levels using the transfection conditions established for the 3T3-L1 adipocytes.

Our results show that lipid-mediated siRNA transfection of fully differentiated adipocytes occurs in suspension with high efficiency as determined by localization of the fluorescent-tagged siRNA in the adipocyte cytoplasm and the decrease in the expression level of five independent and specific targets, including the adipocyte-specific PPARγ and a small set of ubiquitin ligases. We conclude that lipid-based siRNA transfection of 3T3-L1 adipocytes and primary human adipocytes in suspension yields gene knockdown results that are valid as a model for loss-of-function studies in fully differentiated adipocytes. While optimization is required for siRNA-based transfection of any cell type, transfection of adipocytes with siRNA by this method is economical, highly efficient, has a simple workflow, and allows standardization of the ratio of siRNA/cell number, making this approach well-suited for high-throughput screening of fully differentiated adipocytes. In these experiments, we used transfection reagents from a limited number of suppliers, but anticipate that other lipid-based transfection reagents will also provide efficient siRNA transfection of the adipocytes in suspension.
